# The Anticancer Effects of the Garlic Organosulfide Diallyl Trisulfide through the Attenuation of B[a]P-Induced Oxidative Stress, AhR Expression, and DNA Damage in Human Premalignant Breast Epithelial (MCF-10AT1) Cells

**DOI:** 10.3390/ijms25020923

**Published:** 2024-01-11

**Authors:** Dominique T. Ferguson, Equar Taka, Syreeta L. Tilghman, Tracy Womble, Bryan V. Redmond, Shasline Gedeon, Hernan Flores-Rozas, Sarah L. Reed, Karam F. A. Soliman, Konan J. W. Kanga, Selina F. Darling-Reed

**Affiliations:** 1 Pharmaceutical Sciences Division, College of Pharmacy and Pharmaceutical Sciences, Florida A&M University, Tallahassee, FL 32307, USA; dominique3.ferguson@famu.edu (D.T.F.); equar.taka@famu.edu (E.T.); syreeta.tilghman@famu.edu (S.L.T.); tracy.womble@famu.edu (T.W.); shasline1.gedeon@famu.edu (S.G.); hernan.floresrozas@famu.edu (H.F.-R.); sarah1.reed@famu.edu (S.L.R.); karam.soliman@famu.edu (K.F.A.S.); 2 Department of Neuroscience, University of Rochester Medical Center, Rochester, NY 14642, USA; bryan_redmond@urmc.rochester.edu; 3 Department of Biomedical Sciences, College of Medicine, Florida State University, Tallahassee, FL 32306, USA; kwk06@fsu.edu

**Keywords:** diallyl trisulfide, phytochemicals, nutraceuticals, organosulfide, chemoprevention, antitumor, oxidative stress, DNA repair, cancer

## Abstract

Benzo[a]pyrene (B[a]P) is the most characterized polycyclic aromatic hydrocarbon associated with breast cancer. Our lab previously reported that the organosulfur compound (OSC), diallyl trisulfide (DATS), chemoprevention mechanism works through the induction of cell cycle arrest and a reduction in oxidative stress and DNA damage in normal breast epithelial cells. We hypothesize that DATS will inhibit B[a]P-induced cancer initiation in premalignant breast epithelial (MCF-10AT1) cells. In this study, we evaluated the ability of DATS to attenuate B[a]P-induced neoplastic transformation in MCF-10AT1 cells by measuring biological endpoints such as proliferation, clonogenicity, reactive oxygen species (ROS) formation, and 8-hydroxy-2-deoxyguanosine (8-OHdG) DNA damage levels, as well as DNA repair and antioxidant proteins. The results indicate that B[a]P induced proliferation, clonogenic formation, ROS formation, and 8-OHdG levels, as well as increasing AhR, ARNT/HIF-1β, and CYP1A1 protein expression compared with the control in MCF-10AT1 cells. B[a]P/DATS’s co-treatment (CoTx) inhibited cell proliferation, clonogenic formation, ROS formation, AhR protein expression, and 8-OHdG levels compared with B[a]P alone and attenuated all the above-mentioned B[a]P-induced changes in protein expression, causing a chemopreventive effect. This study demonstrates, for the first time, that DATS prevents premalignant breast cells from undergoing B[a]P-induced neoplastic transformation, thus providing more evidence for its chemopreventive effects in breast cancer.

## 1. Introduction

The therapeutic properties of garlic (*Allium sativum*) have been leveraged by many cultures since the beginning of time. The garlic panacea plant, notably, was utilized by the ancient Egyptians, Chinese, Indians, Romans, and Greeks for its many health benefits [1,2,3]. Supplying anticancer phytochemicals and having minimal adverse effects on the human body, diets incorporating garlic reduce the risk of cancer, specifically breast, prostate, colon, and gastrointestinal [4,5,6,7,8,9,10]. Modern research has linked the health benefits of garlic to its anticancer, antioxidant, and antiviral effects, which ultimately enhance the global immune response. These health benefits are primarily associated with garlic’s organosulfur compounds (OSCs), diallyl sulfide (DAS), diallyl disulfide (DADS), and diallyl trisulfide (DATS) [9,11,12]. Previous studies have shown that OSCs modulate cell signaling pathways to control cellular proliferation, providing anticancer effects and strong chemoprevention properties [13,14]. Various studies have proposed the mechanisms involved to explain the cancer-preventive effects of OSCs, including controlling DNA repair mechanisms, cell cycle regulation, the inhibition of DNA adduct formation, mutagenesis, free-radical formation, and tumor growth resulting in garlic’s anti-proliferative effect [14,15,16].

The OSC, DATS, is produced once the garlic bulb is crushed, ground, or cut, which induces the release of alliinase, an enzyme that converts alliin to allicin [1]. DATS makes up approximately 14.6% of the several polysulfides that allicin is converted to and is the most abundant OSC found in fresh garlic oil, with a quantity of roughly 1000 μg per gram of garlic bulb, representing up to 35–60% of garlic oil [1,2,3,4]. DATS is known to have anticancer properties against tumor growth through various mechanisms such as the inhibition of cancer cell proliferation, the inhibition of tumor cell invasion, metastasis, angiogenesis under redox control, the induction of apoptosis, cell cycle arrest, and the inhibition of reactive oxygen species (ROS) in many cancers. Thus, the many anticancer properties of garlic reinforce DATS as a potential chemotherapeutic and chemopreventive agent.

Benzo[a]pyrene (B[a]P) is a ubiquitous, environmental polycyclic aromatic hydrocarbon (PAH) produced naturally through the incomplete combustion of organic material. This compound is responsible for altered epigenetic changes, genotoxic effects in humans and animals, and disordered metabolic changes [5,6]. Several studies have demonstrated that B[a]P exposure may play a role in breast cancer progression, leading to tumor growth and inciting a metastatic cascade [7]. Carcinogenic compounds, such as B[a]P, cause DNA strand breaks, DNA adducts, deletions, mutations, and ROS formation, resulting in genomic instability and abnormalities that may induce carcinogenesis and the development of malignancies. This explains the range of genomic aberrations and diversity in breast cancer [8].

Our lab and others have previously reported that DATS can suppress carcinogenic activity in normal breast and breast cancer cells by inducing cell cycle arrest and apoptosis while also inhibiting ROS formation, DNA damage, and cell proliferation [9,11]. However, DATS’s inhibition of B[a]P-induced neoplastic transformation in premalignant breast cells has not been explored. We hypothesize that DATS will inhibit B[a]P-induced cancer initiation in premalignant breast epithelial (MCF-10AT1) cells. In this study, we evaluated the ability of DATS to attenuate B[a]P-induced oxidative stress and damage through changes in proliferation, clonogenicity, the formation of reactive oxygen species (ROS), 8-hydroxy-2-deoxyguanosine (8-OHdG) levels, and the expression of metabolic, antioxidant, DNA damage, and DNA repair proteins in these premalignant breast epithelial cells. This research uncovers a new and innovative approach to evaluating DATS’s attenuation of chemically B[a]P-induced precancerous transformation in a premalignant human breast epithelial cell line.

## 2. Results

### 2.1. DATS Elicited a Cytotoxic Decrease and B[a]P Increases Cell Growth of MCF-10AT1 Cells

**Figure 1:**
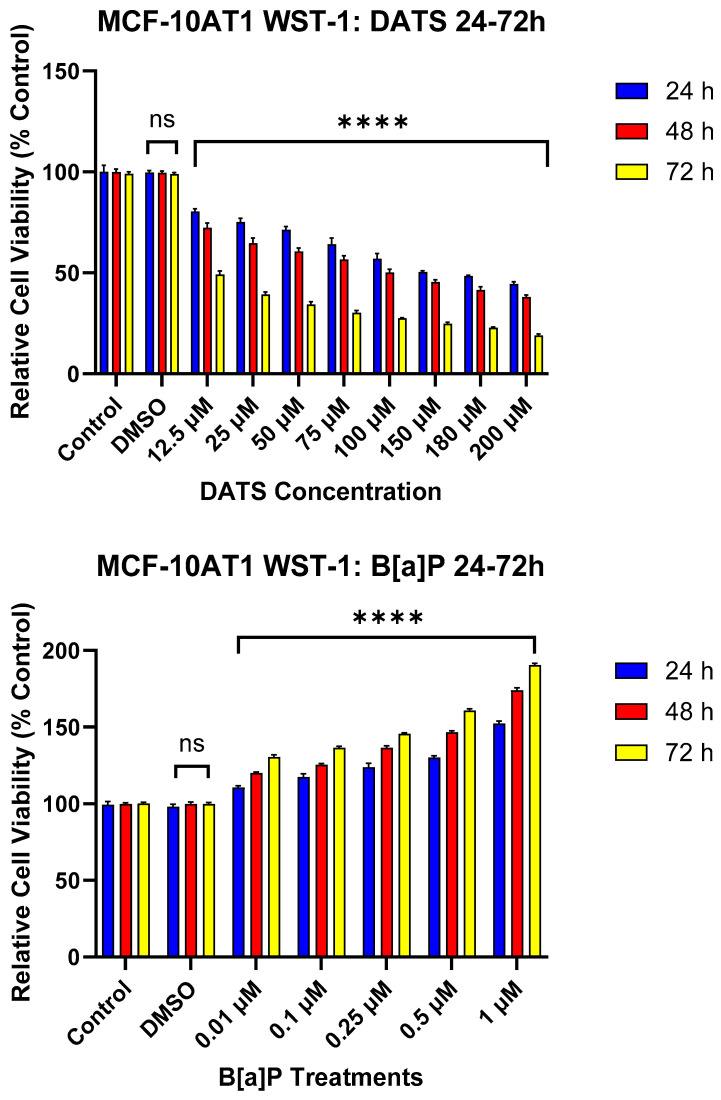
The Effect of DATS and B[a]P on the Viability of MCF-10AT1 Premalignant Breast Epithelial Cells. MCF-10AT1 cells were treated with 0–200 μM DATS or 0.01–1 μM B[a]P for 24–72 h. The effect of DATS had a significant effect between 12.5 and 200 μM. Treatment with 12.5 μM DATS and above caused a significant decrease in cell viability at all time points of exposure compared with the control. Treatment with 0.01 μM B[a]P and above caused a significant increase in cell viability compared with the control. The graph displays all experiments conducted in *n* = 8 and averaged for three biological replicates. The average values ± SEM indicate the results to determine significant differences using one-way analysis of variance (ANOVA) followed by Dunnett’s Multiple Comparison Test between the vehicle control and various treatment groups. (ns indicates no significance and **** *p* < 0.0001).

DATS and B[a]P individual effects at various concentrations were investigated in the MCF-10AT1 cell line after 24, 48, and 72 h. The viability results showed both concentration- and time-dependent decreases in cell viability following DATS treatment in MCF-10AT1 cells over 72 h (Figure 1). Cells treated with DATS showed a significant effect (*p* < 0.0001) between 12.5 and 200 μM DATS when compared with the control. Cell viability significantly decreased following 24 h exposure to 12.5 μM and above of DATS when compared with the control. After 24, 48, and 72 h of treatment, the LC_50_ was 59.08 ± 0.37 μM, 24.06 ± 0.78 μM, and 7.91 ± 0.21 μM, respectively. Similarly, the viability results were concentration- and time-dependent following B[a]P treatment in MCF-10AT1 cells (Figure 1). Treatment with B[a]P concentrations equal to or higher than 0.01 μM significantly increased (*p* < 0.0001) cell viability at 24–72 h of exposure relative to the vehicle control. A treatment of 1 μM B[a]P showed the most significant increase in cell viability relative to the vehicle control (Figure 1). The data from cell viability assays were used to establish DATS’s cytotoxicity and B[a]P concentrations for further studies.

### 2.2. DATS Inhibits B[a]P-Induced Cell Proliferation of MCF-10AT1 Cells Based on BrdU Proliferation Assay 

**Figure 2:**
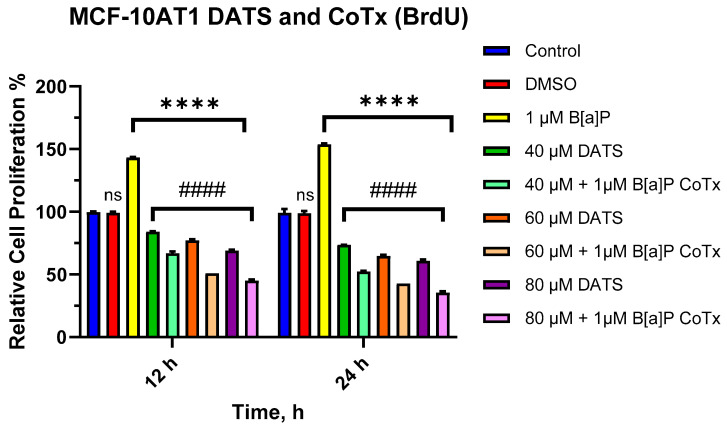
Cell Proliferation Percentage of MCF-10AT1 Cells Treated with B[a]P and DATS. MCF-10AT1 cells were treated with 1 μM B[a]P only, 40–80 μM DATS only, or 1 μM B[a]P + 40–80 μM CoTx for 12 and 24 h. The graph displays all experiments conducted in *n* = 8 and averaged for three biological replicates. The average values ± SEM display the results to determine significant differences using one-way analysis of variance (ANOVA) followed by Dunnett’s Multiple Comparison Test between the vehicle control and various treatment groups. (ns indicates no significance, **** *p* < 0.0001 compared with the control, and #### *p* < 0.0001 when compared with B[a]P treatment).

The BrdU proliferation assay was used to further assess the effect of DATS and/or B[a]P on cell proliferation over a 12–24 h period. The effects of B[a]P and various CoTx were concentration- and -time-dependent. Exposure to 1 μM B[a]P caused a significant increase in cell proliferation at 12 (*p* < 0.0001) and 24 (*p* < 0.0001) h when compared with the vehicle control (Figure 2). There was a significant decrease (*p* < 0.0001) in cell proliferation following DATS (40, 60, and 80 μM) treatments when compared with both the vehicle control and B[a]P alone at 12 and 24 h. Additionally, the CoTx (40–80 μM) also significantly (*p* < 0.0001) decreased cell proliferation when compared with the 1 μM B[a]P and vehicle control, respectively. 

### 2.3. DATS Inhibits B[a]P-Induced Colony Formation of MCF-10AT1 Cells

**Figure 3:**
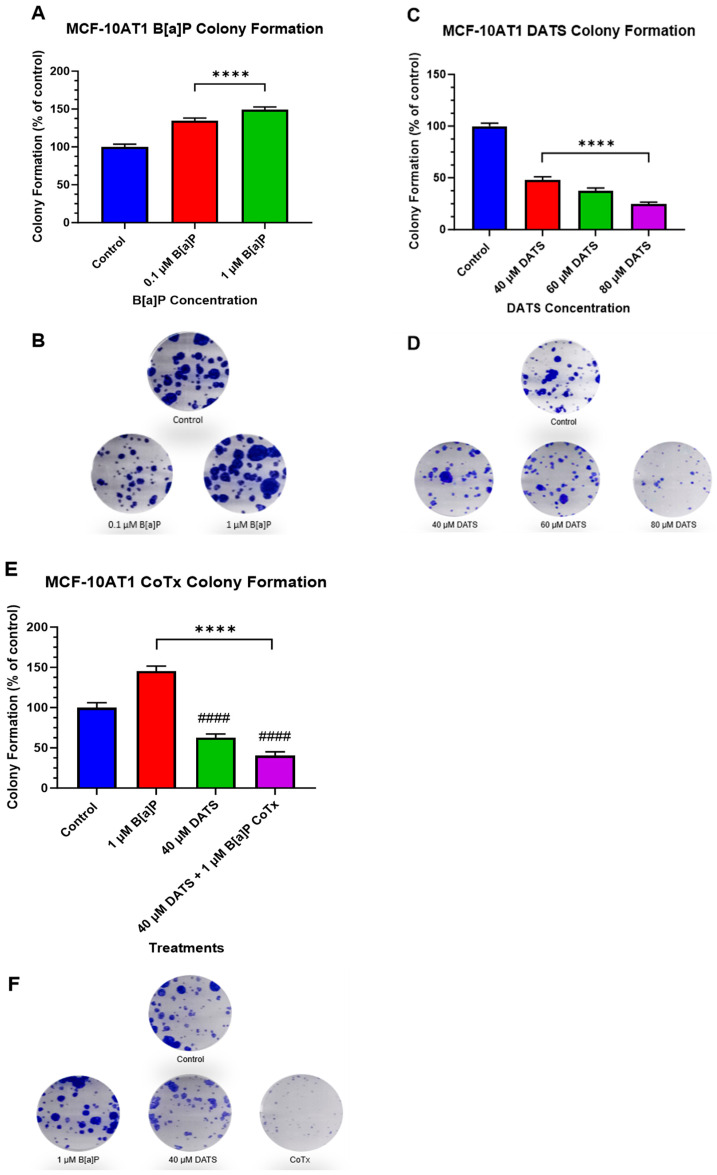
Clonogenic Formation of MCF-10AT1 Cells Treated with B[a]P, DATS, and DATS CoTx. (**A**), Effects of B[a]P on colony formation on MCF-10AT1. (**C**) Effects of DATS on colony formation on MCF-10AT1 cells. (**E**), Effects of 1 μM B[a]P alone, 40 μM DATS alone, and 40 μM CoTx on colony formation on MCF-10AT1 cells. Cells were placed in phenol red-free DMEM supplement with 5% dextran-coated charcoal-treated HS for 24 h before plating. Then 250 cells/well were plated in six-well plates. Seven days later, cells were treated with 0.1% DMSO vehicle control. (**B**,**D**,**F**) and graphs display all experiments conducted in *n* = 3 and averaged for three biological replicates. The average values ± SEM display the results to determine significant differences using one-way analysis of variance (ANOVA) followed by Dunnett’s Multiple Comparison Test between the vehicle control and various treatment groups. (ns indicates no significance, **** *p* < 0.0001 compared with the control, and #### *p* < 0.0001 when compared with B[a]P treatment).

The clonogenic formation assay was used to examine the ability of a single adherent cell treated with B[a]P and/or DATS to survive over time and undergo clonogenic expansion (Figure 3A–F). MCF-10AT1 cells were treated with B[a]P (0.1 and 1 μM), DATS (40, 60, and 80 μM), or CoTx (40 μM DATS + 1 μM B[a]P). The control showed a significant formation of colonies. Treatment with B[a]P significantly increased (*p* < 0.0001) the number of colonies by 35% and 49% for 0.1 μM and 1 μM when compared with the control (Figure 3A,B). Treatments of 40 (*p* < 0.0001), 60 (*p* < 0.0001), and 80 (*p* < 0.0001) μM DATS significantly decreased colony formation in a concentration-dependent manner at 52%, 63%, and 75%, respectively, when compared with the vehicle control (Figure 3C,D). Additionally, we assessed the clonogenic formation of MCF-10AT1 cells treated with 1 μM B[a]P, 40 μM DATS, or CoTx. Treatment with 1 μM B[a]P significantly increased (*p* < 0.0001) the number of colony formations, reaching a maximum of approximately 45% above control levels. Treatment with 40 μM DATS alone significantly decreased (*p* < 0.0001) the number of colony formations by 37% compared with the vehicle control and also significantly reduced (*p* < 0.0001) colony formation by 83% when compared with the 1 μM B[a]P. Treatment with 40 μM CoTx significantly reduced (*p* < 0.0001) the number of colony formations by 60% when compared with the control and 3-fold lower when compared with the 1 μM B[a]P (Figure 3E,F). Furthermore, 40 μM CoTx decreased colony formation compared with 1 μM B[a]P and vehicle control alone.

### 2.4. Reduction of ROS in B[a]P-Treated MCF-10AT1 Cells by DATS

**Figure 4:**
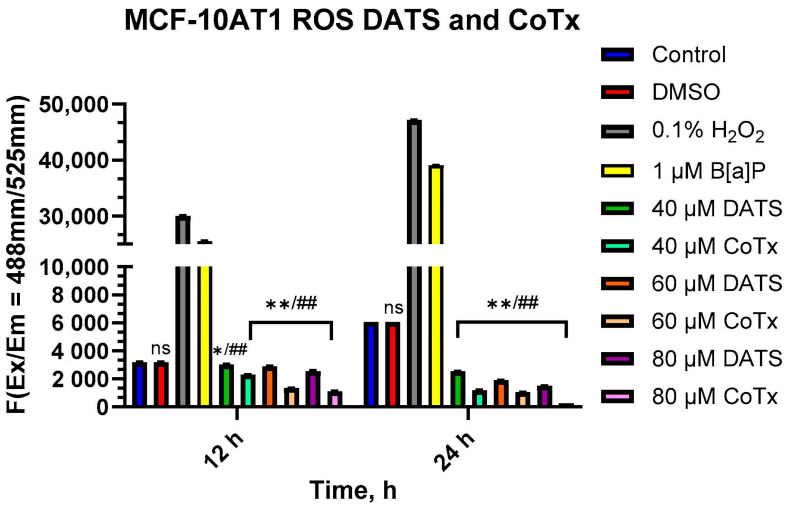
DATS Inhibition of B[a]P-induced ROS in MCF-10AT1 Cells. The cells analyzed for ROS production were treated with B[a]P, DATS, or CoTx for 12 and 24 h and 0.1% hydrogen peroxide was used as a positive control. The graphs display all experiments conducted in *n* = 3 and averaged for three biological replicates. The average values ± SEM display the results to determine significant differences between the vehicle control and various treatment groups. (ns indicates no significance, * *p* < 0.05, ** *p* < 0.01 compared with the control, and ## *p* < 0.01 when compared with B[a]P treatment).

To measure oxidative stress, MCF-10AT1 cells treated with DATS and B[a]P for 12 and 24 h periods were measured for levels of ROS (Figure 4). B[a]P caused a significant increase in ROS production, which peaked at 24 h. All treatments with DATS and CoTx (40–80 μM) concentrations exhibited a concentration- and time-dependent response for 12 and 24 h with an overall decrease in ROS production. MCF-10AT1 cells treated after 12 h with 40 μM DATS (*p* < 0.05) and 60–80 μM DATS (*p* < 0.01) indicated a significant decrease in ROS production by 33%, 35%, and 39%, respectively, compared with the control. Similarly, MCF-10AT1 cells treated after 24 h with 40–80 μM DATS (*p* < 0.01) also decreased ROS production by 39%, 52%, and 67% compared with the vehicle control. The 12 h CoTx also significantly decreased (*p* < 0.01) B[a]P-induced ROS by 44%, 74%, and 90% at 40, 60, and 80 μM, respectively. For 24 h, CoTx significantly decreased (*p* < 0.01) B[a]P-induced ROS by 83%, 95%, and 98%, respectively, at 40, 60, and 80 μM. When compared with the 1 μM B[a]P, all the treatments also indicated a significant decrease (*p* < 0.01) in ROS production. As detected by the ROS assay, these results indicate that all the treatments with DATS and CoTx effectively inhibited ROS formation. 

### 2.5. Inhibition of B[a]P-Induced Oxidative (8-OHdG) DNA Damage by DATS in MCF-10AT1 Cells

**Figure 5:**
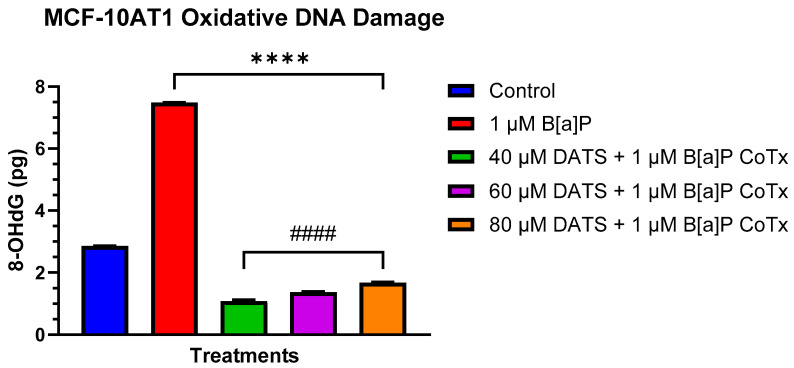
DNA Damage Detection of MCF-10AT1 Cells Treated with DATS and/or B[a]P. MCF-10AT1 cells were treated with 1 μM B[a]P only or 1 μM B[a]P + 40–80 μM CoTx for 24 h. The graph displays 8-OHdG (picogram (pg) levels) as an indicator of oxidative DNA damage. The graph displays all experiments conducted in *n* = 8 and averaged for three biological replicates. The average values ± SEM display the results to determine significant differences using a *t*-test between the vehicle control and various treatment groups. (ns indicates no significance, **** *p* < 0.0001 compared with the control, and #### *p* < 0.0001 when compared with B[a]P treatment).

The Epiquik 8-OHdG DNA Damage Quantification Direct Kit was used to measure oxidative DNA damage levels. B[a]P caused a significant increase (*p* < 0.0001) in 8-OHdG when compared with the vehicle, thereby considerably increasing oxidative DNA damage. All treatments with DATS and CoTx (40–80 μM) concentrations exhibited a concentration-dependent response with an overall significant decrease (40 μM CoTx *p* < 0.0001, 60 μM CoTx *p* < 0.0001, and 80 μM CoTx *p* < 0.0001) of 8-OHdG when compared with the control; all CoTxs also significantly decreased (*p* < 0.0001) 8-OHdG, indicating a reduction in oxidative DNA damage and oxidative stress when compared with the 1 μM B[a]P (Figure 5). While the 8-OHdG levels of CoTx were significantly decreased, the 8-OHdG levels increased with increasing concentrations of DATS in the CoTx.

### 2.6. DATS Attenuates B[a]P-Induced Hypoxic Conditions under Acute Response in Premalignant MCF-10AT1 Cells

Tumor growth is associated with cellular proliferation and the subsequent oxygen deprivation of the microenvironment [17,18]. Hypoxic proteins are key regulators for cells to adapt, overcome low oxygen, and maintain oxygen homeostasis. The Aryl hydrocarbon Receptor (AhR) is a ligand-activated transcription factor that influences tumorigenesis by mediating carcinogenic toxicity through direct binding to environmental contaminants such as B[a]P. The hypoxia-inducible factor-1beta (HIF-1β)/aryl hydrocarbon receptor translocator (ARNT) is a transcription factor that controls adaptive responses from oxidative stress as an indicator of hypoxic/acute/environmental stress response. Additionally, B[a]P induces the cytochrome P450 enzyme, CYP1A1, a major contributor to PAH metabolism, inducing AhR binding to increase ROS generation and DNA adduct formation resulting in oxidative stress.

**Figure 6:**
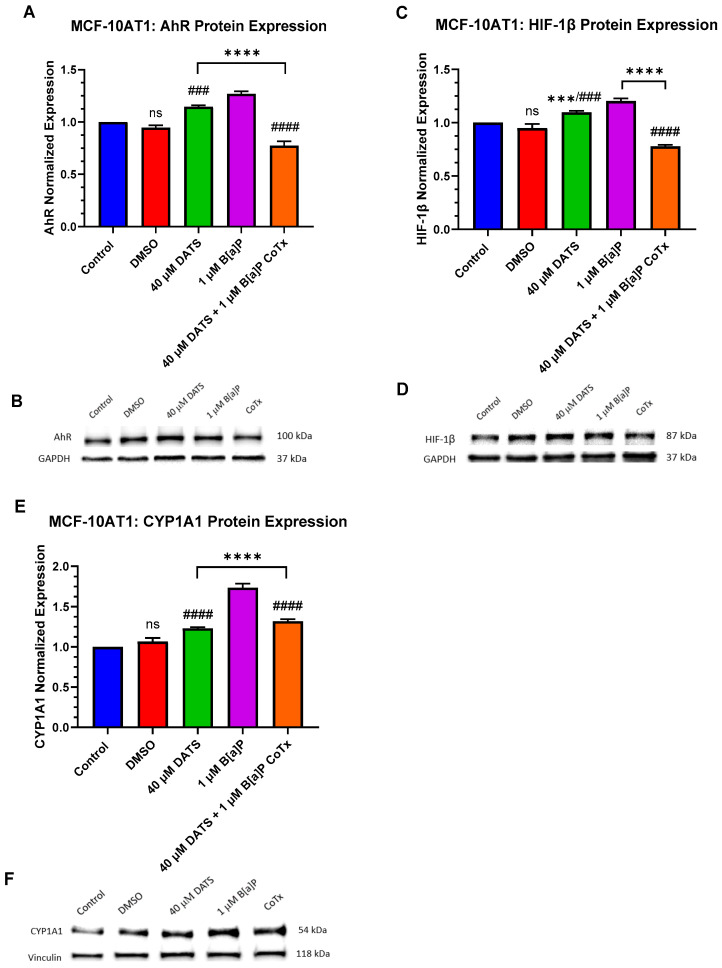
AhR, HIF-1β, and CYP1A1 Expression in Premalignant (MCF-10AT1) Breast Epithelial Cells. AhR, HIF-1β, and CYP1A1 protein expression were normalized and measured using densitometry (**A**–**F**). The immunoblots represented the protein expression after 24 h-post treatment for AhR, HIF-1β, and CYP1A1. The graph displays all experiments conducted in *n* = 3 and averaged for three biological replicates. The average values ± SEM display the results to determine significant differences using one-way analysis of variance (ANOVA) followed by Dunnett’s Multiple Comparison Test between the vehicle control and various treatment groups. (ns indicates no significance, *** *p* < 0.001, **** *p* < 0.0001 compared with the control and ### *p* < 0.001, #### *p* < 0.0001 when compared with B[a]P treatment).

AhR expression was evaluated for changes in protein expression following 24 h exposure to 1 μM B[a]P, 40 μM DATS, and 40 μM CoTx. GAPDH loading control was used to normalize the protein expression of all the treatments. All the treatments were compared with a control and the 1 μM B[a]P treatment (Figure 6A,B). In the MCF-10AT1 cells, AhR expression was significantly increased when exposed to 1 μM B[a]P (*p* < 0.0001) or 40 μM DATS (*p* < 0.0001) when compared with the control, but the effect of AhR expression on 40 μM DATS-treated cells was significantly decreased (*p* < 0.001) when compared with 1 μM B[a]P. The 40 μM CoTx significantly reduced (*p* < 0.0001) AhR expression when compared with the control, and significantly decreased (*p* < 0.0001) AhR expression when compared with the 1 μM B[a]P. Thus, the reduction in AhR expression by 40 μM CoTx was much more prominent in all the treatments compared with B[a]P and the control. The presence of AhR expression was validated in all treatments through Western blot analysis.

HIF-1β/ARNT is induced by B[a]P to cause upregulation in the hypoxic response. HIF-1β expression was evaluated for changes in protein expression following treatment with the control, the 0.1% DMSO vehicle control, 1 μM B[a]P, 40 μM DATS, and 40 μM CoTx in both non-cancerous epithelial MCF-10A, and premalignant MCF-10AT1 cells. All treatments were compared with the vehicle control and the 1 μM B[a]P treatment (Figure 6C,D). In MCF-10AT1 cells, 1 μM B[a]P (*p* < 0.0001) and 40 μM DATS alone (*p* < 0.001) increased HIF-1β expression while CoTx significantly decreased (*p* < 0.0001) HIF-1β expression when compared with the control. The 40 μM DATS alone significantly increased (*p* < 0.001) HIF-1β expression when compared with the B[a]P. Exposure to 40 μM CoTx significantly decreased (*p* < 0.0001) HIF-1β expression when compared with 1 μM B[a]P alone, respectively.

The cytochrome P450 1A1 (CYP1A1) expression is present in MCF-10AT1 cells [19]. CYP1A1 expression was evaluated for changes in protein expression with 1 μM B[a]P, 40 μM DATS, and 40 μM CoTx. Vinculin loading control was used to normalize the protein expression of all the treatments. All treatments were compared with the control and the 1 μM B[a]P treatment (Figure 6E,F). In MCF-10AT1 cells, 1 µM B[a]P, 40 μM DATS, and 40 μM CoTx significantly increased (*p* < 0.0001) CYP1A1 expression when compared with the control. In addition, when compared with B[a]P alone, CYP1A1 expression was significantly decreased (*p* < 0.0001) in the MCF-10AT1 cells exposed to DATS alone. The CoTx significantly attenuated (*p* < 0.0001) B[a]P-induced CYP1A1 expression when compared with B[a]P alone. The results of these experiments indicate that CYP1A1 protein expression appears to be more pronounced following B[a]P treatment in the premalignant MCF-10AT1 cells.

### 2.7. DATS Inhibits B[a]P-Induced DNA Damage and Induces DNA Repair under Acute Response in Premalignant MCF-10AT1 Cells

The DNA damage response pathway, base excision repair (BER), utilizes 8-oxoguanine DNA glycosylase (OGG1) to detect and remove single base DNA damage and DNA polymerase beta (POLβ) to resynthesize the single-strand break. BER repairs DNA damage caused by oxidation or alkylating adducts to maintain genetic stability and prevent DNA damage tolerance dysregulation and cancer progression [20,21].

**Figure 7:**
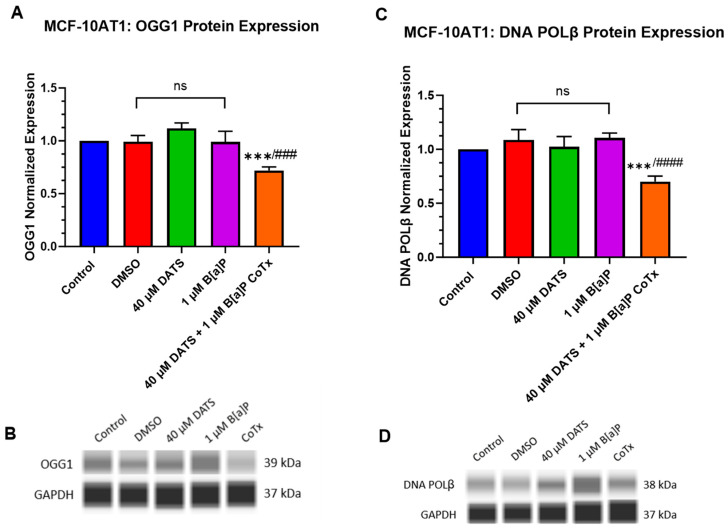
Expression of OGG1 and POLβ in Premalignant (MCF-10AT1) Breast Epithelial Cells. OGG1 and POLβ protein expression was normalized and measured using densitometry (**A**–**D**). The protein expression of OGG1 and POLβ was measured using ProteinSimple SW Compass 6.2.0 software. The immunoblots represented the protein expression after 24 h-post treatment for OGG1 and POLβ. The graph displays all experiments conducted in *n* = 3 and averaged for three biological replicates. The average values ± SEM display the results to determine significant differences using one-way analysis of variance (ANOVA) followed by Dunnett’s Multiple Comparison Test between the vehicle control and various treatment groups. (ns indicates no significance, *** *p* < 0.001 compared with the control and ### *p* < 0.001, #### *p* < 0.0001 when compared with B[a]P treatment).

B[a]P induces oxidative DNA damage through the induction of ROS generation. This oxidative damage can be repaired by inducing the OGG1 repair enzyme. OGG1 was evaluated for changes in protein expression following treatment with 1 μM B[a]P, 40 μM DATS, and 40 μM CoTx in MCF-10AT1 cells. GAPDH loading control was used to normalize the protein expression of all the treatments. All the treatments were compared with the control and the 1 μM B[a]P treatment (Figure 7A,B). Exposure to 40 μM CoTx significantly decreased OGG1 protein expression when compared with the control (*p* < 0.001) and 1 μM B[a]P alone (*p* < 0.0001) (Figure 7A,B).

The POLβ enzyme can repair ROS-induced oxidative DNA damage. POLβ expression was evaluated for changes in protein expression following treatment with 1 μM B[a]P, 40 μM DATS, and 40 μM CoTx in the MCF-10AT1 cell line. GAPDH loading control was used to normalize the protein expression of all the treatments. All the treatments were compared with the control and the 1 μM B[a]P treatment (Figure 7C,D). In MCF-10AT1 cells, exposure to 40 μM CoTx significantly decreased (*p* < 0.001) POLβ protein expression when compared with the control and significantly reduced (*p* < 0.0001) POLβ expression when compared with 1 μM B[a]P alone, respectively (Figure 7C,D). Exposure to 1 μM B[a]P and 40 μM DATS induced no significant changes compared with the control.

## 3. Discussion

B[a]P is a first-class ubiquitous environmental pollutant and a reproductive and developmental toxicant formed primarily by the incomplete combustion of carbon-containing fuels [22,23]. Epidemiological evidence has confirmed that increased rates of breast cancer are associated with exposure to high levels of B[a]P [23]. In vivo and in vitro studies have shown that the mechanism of B[a]P-induced breast cancer may involve DNA damage, DNA mismatch repair, DNA adduct formation, and ROS formation, exhibiting its effects of tumor initiation and malignant transformation in human mammary gland tissue [22,23]. 

Nutraceuticals, such as the bioactive compounds found in garlic (*Allium sativum*), have potential health-associated benefits, including reducing high blood pressure, improving cholesterol levels, and amplifying the immune system [24]. OSCs such as allicin, the primary bioactive compound in garlic, play a significant role in garlic’s health-associated benefits due to its many protective medicinal properties, including anticancer, anti-inflammatory, antimicrobial, cardioprotective, antidiabetic, and antioxidant effects [24]. However, the overall anticancer properties of OSCs have not been fully elucidated. The proposed mechanism of the anticancer potential of garlic and its bioactive OSC derivatives lies in modulating various signaling pathways, leading to its chemopreventive, antiproliferative, anti-inflammatory, and antioxidant effects [24]. Our lab and others have previously reported that DATS affects chemical-induced carcinogenesis by suppressing ROS formation and the induction of cell cycle arrest in normal epithelial and cancer cells [9,12,25,26]. However, there are no in vitro studies examining the impact of DATS on breast cancer progression as epithelial cells transition through a multiyear, multistep, multiscale, and multipath process to a cancerous phenotype. To address this deficit in the literature, we used the *Ha-ras*-transfected premalignant MCF-10AT1 cell line as a model to evaluate neoplastic transformation. This cell line is known to produce lesions, thus generating carcinomas that resemble atypical hyperplasia and carcinoma in situ in women [27,28]. While it has been established in a previous study [19] that long-term exposure to B[a]P enhances the cancerous phenotype in this transformed early-stage progression model, no documented data have examined garlic’s impact on chemical-induced neoplastic transformation using this cell line. To gain more insight into the effects of the garlic OSC DATS on B[a]P-induced cancer as it progresses from an epithelial to a cancerous phenotype, our lab used this MCF-10AT1 cell line. Thus, the focus of this study was to evaluate how B[a]P-induced activities can be attenuated by the OSC, DATS, through alterations in cell proliferation, clonogenic formation, the formation of damaging ROS that can lead to DNA damage, and the interplay between various proteins expressed (AhR, ARNT/HIF-1β, CYP1A1, OGG1, and DNA POLβ) as indicators of DNA damage which may lead to the neoplastic transformation of B[a]P-treated premalignant breast epithelial MCF-10AT1 cells.

Previous studies in our lab and others have shown that DATS effectively inhibits carcinogen-induced cellular damage in normal epithelial and cancer cells [9,29,30]. However, there is a paucity of information concerning the impact of DATS on cell viability, proliferation, and clonogenic formation in carcinogen-induced premalignant breast cells. The DATS and B[a]P concentrations used in the experiments of this study were chosen based on established exposures/physiological concentrations and previous studies performed in our lab [9,11,31,32]. DATS is the most potent organosulfide and studies published by other researchers [31,33,34] used lower concentrations of 20 μM and 40 μM of DATS since 40 μM is comparable to those used in animal studies. Following our review of previous studies and our data, we decided to use the lower concentration of 40 μM since the results were very similar to 60 μM DATS. Additionally, a review of the cell viability studies using normal breast epithelial cells showed that the higher the concentration of DATS, the more cell death occurred in the cells [9]. Our objective was to identify a concentration of DATS to be used as a chemopreventive agent that will cause minimal toxicity in normal epithelial cells. The WST-1 assay was used to assess the cell viability of MCF-10AT1 cells following treatment with DATS or B[a]P. In this study, B[a]P significantly increased cell viability between 0.01 and 1 μM, with a more pronounced effect at 1 μM. Evidence has shown that DATS can suppress viability in various malignancies (breast, prostate, colon, lung, stomach, cervix, and bone) by inducing apoptosis and cell cycle arrest, thus exerting its antitumor effect [14,15,35,36,37]. In these premalignant cells, we found that DATS significantly decreased cell viability between 12.5 and 200 μM in a concentration- and exposure-time-dependent manner. 

While previous studies have shown B[a]P-induced cell proliferation in breast epithelial and cancer cells [38,39,40], there are no documented proliferation studies assessing varying concentrations of B[a]P in these premalignant cells. In vitro and in vivo studies have shown that DATS attenuates chemically induced proliferation in different cancers [30,31,41]. BrdU is a pyrimidine analog incorporated into a newly synthesized DNA [42]. A rapidly proliferating human cell has a total of 24 h to divide within the cell cycle; however, the S phase does not occur until about 11 h, typically depending on the type of cell [43]. Furthermore, a study by Jaio et al. [44] found that increased cyclin D1 levels, indicative of G1-S transition, peaked at 12 h in B[a]P-treated human embryo lung fibroblasts. Since BrdU is incorporated into DNA based on how much is replicated during the S phase of the cell cycle, we decided to assess both 12 and 24 h time points. The BrdU assay was used to determine the impact of the combined treatments of DATS/B[a]P on the proliferation of the MCF-10AT1 cells. These results supported our hypothesis that DATS CoTx(s) effectively inhibited B[a]P-induced cell proliferation at 12 h (*p* < 0.0001) and 24 h (*p* < 0.0001), with a more pronounced effect at 24 h. 

We also assessed clonogenic expansion to measure cell growth and the survival of premalignant cells via the colony formation assay. DATS alone and CoTx were also found to significantly decrease (*p* < 0.0001) clonogenic formation after seven days of treatments, whereas B[a]P significantly increased (*p* < 0.0001) clonogenic formation during the same time point. While there are no studies showing the effect of DATS on clonogenic expansion, others have previously reported the impact of B[a]P in MCF-10AT1 cells [19]. A study performed by Stan et al. [26] reported the impact of DATS on clonogenic formation in ductal carcinoma in situ and minimally invasive breast cancer cells. Cancer can be induced by the gain of function mutations to oncogenes or growth factor signaling pathways that may lead to uncontrolled cell growth or proliferation. A recent case-control study by Kjaer et al. [45] revealed that abnormal pre-treatment serum levels of Epidermal growth factor (EGFR) and its ligands were found in women with early-stage breast cancer. Several studies have shown that B[a]P and its metabolites can promote cell proliferation and tumorigenesis through increases in MAPK and PI3K/AKT/ERK pathways in normal and neoplastic cells [44,45]. Mello et al. [46] revealed that the transfection of the Ha-Ras oncogene in B[a]P-transformed MCF-10F floating breast epithelial cells induces a more aggressive tumorigenic phenotype. We used premalignant MCF-10AT1 cells, transfected with the Ha-Ras oncogene, in our cell viability, proliferation, and colony formation studies. In 2009, research performed by Malki et al. [47]demonstrated that the garlic organosulfide, DATS, induced apoptosis in MCF-7 cells with a reduced effect in MCF-12A normal epithelial cells. DATS affects cell viability, proliferation, and colony formation, most likely due to the cancer initiation induced by the Ha-Ras oncogene transfected in MCF-10AT1 cells. This effect is enhanced when these transfected cells are treated with B[a]P, most likely due to the impact of DATS on the B[a] P-induced mutations generated during the chemical-induced transformation of these cells. DATS’s inhibitory effects on in vivo and in vitro cancer models are much more pronounced than in in vitro chemical-induced epithelial cell models [41,48,49,50,51,52]. When DATS was used in this study, it was an effective attenuator of B[a]P-induced proliferation and clonogenic formation in these premalignant cells. DATS’s significant inhibition of cell viability, cell proliferation, and clonogenic expansion in this study provides new insight into it as an effective inhibitor in preventing premalignant cells from further undergoing B[a]P-induced neoplastic transformation.

B[a]P, a prototype of polycyclic aromatic hydrocarbons (PAHs), is formed as a by-product from various thermal processes, such as the burning of fossil fuels, cigarettes, wood, and organic materials [53,54]. The effects of B[a]P occur through the biotransformation of cytochrome P450 and microsomal epoxide forming the carcinogenic metabolite BPDE where ROS are produced as a by-product and DNA adducts are created, leading to erroneous replication and mutagenesis [54,55,56]. Since it is highly likely that ROS changes may precede growth changes, we aimed to capture this phenomenon by assessing an earlier time point of both 12 and 24 h. In this study, B[a]P significantly increases (*p* < 0.0001) ROS generation, which is a possible indicator of oxidative damage. Increased levels of intracellular ROS may cause DNA damage, leading to mutations and neoplastic transformation from alteration in replication and transcription [57]. Our most recent findings, showing that DATS effectively attenuated B[a]P-induced ROS formation, are also supported by previous studies [12] performed in this lab. Similarly, these studies showed that DATS was effective in attenuating B[a]P-induced lipid peroxide formation. In this study, DATS attenuated ROS and effectively reduced carcinogen-induced free radical induction, thus exhibiting a cytoprotective effect against PAHs in a premalignant cell line.

The aryl hydrocarbon receptor (AhR) pathway mediates toxicity and the tumor-promoting properties of environmental contaminants [58]. B[a]P is a primary ligand of AhR that directly binds to the receptor and induces its biological effects associated with the major stages of tumorigenesis [58,59]. In this study, AhR expression was significantly increased (*p* < 0.0001) following 24 h exposure to 1 μM B[a]P in MCF10AT1 cells. These results were supported by Dononi et al. [19], who recently reported that the mRNA and protein expression of AhR and G-protein coupled receptor 30 (GPR30), both markers of poor prognosis in cancer patients [60,61], were concomitantly expressed following low-dose chronic exposure to B[a]P in these premalignant cells. Their study correlated these cells within a triple negative context and found that low-dose, chronic exposure to B[a]P and/or Bisphenol A (BPA) increased the cancerous properties of the MCF-10AT1 cells. Further, Stanford et al. [62] found that activation of the AhR led to the development of breast epithelial cells with molecular and functional characteristics of cancer stem-like cells. According to Guarnieri et al. [63], higher AhR expression is correlated with a greater expression of genes encoding inflammatory factors and invasive behavior in cancer cells. The CoTx significantly decreased (*p* < 0.0001) the AhR response at the same time point, thus attenuating the AhR expression in B[a]P-treated premalignant breast epithelial cells. These results provide new evidence of DATS’s ability to attenuate chemically induced AhR expression in a premalignant cell model and its chemopreventive potential by inhibiting neoplastic progression.

The aryl hydrocarbon receptor nuclear translocator (ARNT), also known as hypoxia-inducible factor-1beta (HIF-1β), plays a crucial role in regulating tumorigenesis [64]. To become active, AhR must form a heterodimeric complex with ARNT that triggers the transcriptional activation of several target genes, including aldehyde dehydrogenase family 3, subfamily 1 (ALDH3A1), NAD(P)H dehydrogenase quinone (NQO1), glutathione-S-transferase alpha 1 (GSTA1), UDP glucuronosyltransferase family 1 member A6 (UGT1A6), and CYP1A1 and CYP1A2 to form the “AhR gene battery”. In this study, we have demonstrated that ARNT/HIF-1β expression was significantly increased (*p* < 0.0001) following 24 h exposure to 1 μM B[a]P in MCF-10AT1 cells. An increased expression of AhR and ARNT suggests an interaction and formation of an active heterodimeric complex. Several studies [65,66,67,68] have been published concerning the interaction between active AhR and the inducible transcription factor, NF-kappaB, in inflamed stromal and tumoral cells. These studies provide further evidence that active AhR plays a role in cancer progression. CoTx significantly decreased (*p* < 0.0001) ARNT/HIF-1β response at the same time point in B[a]P-treated MCF-10AT1 cells. The results presented in this study align with the reduction in oxidative damage and provide new evidence of DATS’s ability to suppress the expression of AhR and ARNT/HIF-1β when concurrently combined with B[a]P to inhibit further neoplastic transformation.

The cytochrome P450 enzyme, CYP1A1, is a significant contributor to the metabolism of PAHs by inducing AhR through the binding of environmental pollutants, such as B[a]P, leading to the development of tumorigenesis [69,70]. In the current study, CYP1A1 protein expression was significantly increased (*p* < 0.0001) following 24 h exposure to 1 μM B[a]P in the MCF-10AT1 cell line. CYP1A1 expression was significantly increased (*p* < 0.0001) in MCF-10AT1 cells treated with DATS alone or DATS CoTx when compared with the control but decreased (*p* < 0.0001) considerably when compared with the B[a]P. While DATS CoTx reduced AhR, ARNT/HIF-1β, and CYP1A1 expression when compared with B[a]P, the increase in CYP1A1 expression when compared with the control is in alignment with previous studies. Various studies [71,72] show that natural products and phytochemicals may exert their chemopreventive effects by inducing or inhibiting CYP1A1 expression. Studies have shown that the increase in CYP1A1 expression induces ROS formation [73,74,75,76,77]. Thus, the reduction in CYP1A1 expression by DATS in the CoTx premalignant cells may explain the attenuation of ROS production observed in this study. The increase in CYP1A1, AhR, and ARNT/HIF-1β expression observed in the MCF-10AT1 cells exposed to DATS alone when compared with the control is unexplained. DATS may inhibit growth and migration in these premalignant cells in a similar fashion as observed in a study using a newly identified AhR agonist, Flavipin, in triple-negative breast cancer cells [78]. The induction of AhR, ARNT, and CYP1A1 by Flavipin decreased cell migration and invasion in T47D and MDA-MB-231 cells. More studies must be performed to gain a better understanding of the impact of DATS single exposure on these premalignant cells. Based on our previous and current findings [79,80,81,82,83], the reduced expression of AhR, HIF-1β, and CYP1A1 in concert with decreased ROS production and 8-OHdG levels after exposure to the DATS CoTx suggests that natural products like OSCs may exert their chemopreventive effect by competing with PAHs for both AhR and ARNT/HIF-1β receptors. This inhibits CYP1A1 protein expression, thus attenuating B[a]P-induced toxicity in premalignant breast epithelial cells.

The DNA repair system plays a significant role in maintaining cell genomic stability [84]. Tandem mutations, mCG → TT, may be generated through the promotion of the double misincorporation of a single lesion during DNA replication by base substitution errors with adenine instead of cytosine [85]. Previous research in our lab and by others has shown that B[a]P-induced oxidative DNA damage and ROS formation may lead to DNA strand breaks in nontumorigenic breast epithelial cells and human breast cancer cells [12,40,86]. In Nkrumah-Elie et al. [9], the DATS-mediated attenuation of cellular carcinogenesis was shown in B[a]P-induced normal breast epithelial MCF-10A cells by mechanisms including lipid peroxide production, DNA strand break formation, and cell cycle arrest. Outside of the research performed in our lab, few studies have evaluated OSCs, specifically DATS, and their role in inhibiting DNA strand breaks through the activation of DNA repair. In this study, 1 μM B[a]P significantly increased (*p* < 0.0001) 8-OHdG, an indicator of induced oxidative DNA damage and stress, in a premalignant breast epithelial cell line. Our findings indicate that varying concentrations of DATS ranging from 40 to 80 µM with 1 µM B[a]P co-treated significantly attenuated (*p* < 0.0001) B[a]P-induced increases in 8-OHdG levels in premalignant breast epithelial cells, thus indicating a suppression of oxidative DNA damage and stress. This study investigated the most effective concentration, 40 μM CoTx, in inhibiting B[a]P-induced DNA damage. The data presented in this study of DATS-induced reduction in oxidative stress correlates with the decrease in 8-OHdG levels. Therefore, DATS can alleviate intracellular ROS and DNA damage, thus exerting a chemopreventive effect and preventing neoplastic transformation. 

BER is a major genome maintenance pathway that uses OGG1 to recognize and remove 8-oxo-7,8-dihydroguanine (8-oxoG) from oxidative DNA damage to prevent genomic instability [87]. DNA polymerase β (POLβ) is recruited to fill the single gap caused by the DNA glycosylase-initiated removal of 8-oxo-G with guanine to repair lesion damage from ROS and alkylating agents [88,89]. These experiments demonstrated that B[a]P had no effect while the 40 μM DATS CoTx significantly decreased (*p* < 0.001) OGG1 and POLβ protein expression in premalignant MCF-10AT1 cells. The inhibition of OGG1 and POLβ with 40 μM CoTx suggests that the observed decrease in 8-OHdG levels and oxidative DNA damage may occur through another mechanism in these transforming premalignant breast epithelial cells. The results from this research suggest that DATS CoTx may prevent further oxidative damage while inhibiting OGG1 and POLβ DNA repair mechanisms, thus allowing premalignant cells to undergo cell death and prevent B[a]P-induced cancerous transformation. Further studies must be performed to gain a better understanding of the underlying mechanisms of DATS on chemically induced DNA damage and subsequent cancer progression in these cells. 

## 4. Materials and Methods

### 4.1. Cell Line, Chemicals, and Reagents

MCF-10AT1 cells were acquired from the Animal Model and Therapeutic Core (AMTEC) Barbara Ann Karmanos Cancer Institute, Wayne State University (Detroit, MI, USA). Dulbecco’s Modified Eagle Medium/Nutrient Mixture F-12 (DMEM/F-12) phenol red-free media, Hanks Balanced Salt Solution (HBSS), Phosphate-Buffered Saline (PBS), 10X Trypsin in HBSS, hydrocortisone, HEPES, calcium chloride, epidermal growth factor, horse serum, human insulin (Novolin R), and penicillin/streptomycin were purchased from Thermo Fisher Scientific (Wilmington, DE, USA). DATS (99.2% purity, 200 mM stock) was obtained from LKT Laboratories (St. Paul, MN, USA) and dissolved in dimethyl sulfoxide (DMSO) (200 mM stock). B[a]P (10 mM stock), DMSO, The CELLPRO-RO Roche Cell Viability and Proliferation Reagent WST-1, and all other chemicals were purchased from Sigma-Aldrich (St. Louis, MO, USA) and stored at −20 °C. The Bromodeoxyuridine (BrdU) Cell Proliferation Assay kit was acquired from Cell Signaling Technology (Danvers, MA, USA). The Reactive Oxygen Species (ROS) Detection Assay Kit was purchased from BioVision Incorporated (Milpitas, CA, USA). The EpiQuik-8-OHdG DNA Damage Quantification Direct Kit (Colorimetric) was purchased from EpiGentek (Farmingdale, NY, USA). The Qiagen Genomic-tip 20/G, Genomic DNA buffer set, and proteinase k were obtained from Qiagen (Germantown, MD, USA). The primary antibodies used were anti-DNA polymerase β (ab26343) and anti-Ogg1 (ab62826) purchased from Abcam (Boston, MA, USA), and the loading control GAPDH mAb (#D16H11) was purchased from Cell Signaling (Danvers, MA, USA). The anti-Erk1 primary antibody, HeLa lysate controls, Anti-Rabbit Detection Module, 8 × 25 capillary cartridges, and 12–230 Separation Module were purchased from ProteinSimple (San Jose, CA, USA). 

### 4.2. Cell Model and Culture

MCF-10AT1, previously known as MCF10AneoT cells, are derived from the MCF-10 human breast epithelial model system. MCF-10AT1 cells are transfected with T24 *Ha-ras*, derived from xenograft-passed MCF10AneoT cells in immune-deficient mice and are shown to produce lesions resembling atypical hyperplasia and carcinoma in situ in women [27,28]. These lesions generate carcinomas and can progress into neoplastic transformation [28]. This model highlights neoplastic transformation in a transformed *ras*-transfected premalignant cell line.

MCF-10AT1 cells were cultured in phenol red-free DMEM/F12 media containing calcium chloride (1.05 mM), epidermal growth factor (20 ng/mL), horse serum (5%) 1% penicillin (100 U/mL)/streptomycin (0.1 mg/mL), human insulin (10 μg/mL), HEPES (1M), and hydrocortisone (0.5 μg/mL). Cells were housed in a humidified incubator at 37 °C, 5% CO_2_, and allowed to grow to 75–90% confluency. The media were replaced every 2–3 days, and the cells were sub-cultured every 5 days.

### 4.3. Cell Treatments

MCF-10AT1 cells were cultured and divided into the following distinct treatment groups: (1) 40, 60, or 80 μM of DATS, (2) 1 μM of B[a]P, or (3) B[a]P and DATS co-treatments (CoTx), consisting of 40, 60, or 80 μM of DATS and 1 μM B[a]P, concurrently treated. The cell viability studies were conducted utilizing cells treated with or without DATS (12.5, 25, 50, 75, 100, 150, 180, and 200 μM) and with or without B[a]P (0.01, 0.1, 0.25, 0.5, and 1) for 24, 48, and 72 h. The Lethal Concentration 50 (LC_50_) was determined by logistic regression analysis using GraphPad Prism 9.0 software (San Diego, CA, USA). The clonogenic formation studies were completed with cells treated with (0.01 and 1 μM) B[a]P, (40, 60, and 80 μM DATS) or CoTx with (1 μM) B[a]P and (40, 60, or 80 μM) DATS for 7 days. The cells were prepared under low light conditions in media for all experiments and treatments, employing 0.1% DMSO as the vehicle control. Once treated, the cells were placed in a humidified incubator and cultured for 12 or 24 h at 37 °C, 5% CO_2_. After undergoing treatment, the adherent cells were subjected to trypsinization, collected, and centrifuged at 1200 rpm for 5 min. Subsequently, the cell pellets were reconstituted by resuspension in PBS devoid of Mg^2+^ or Ca^2+^. 

### 4.4. Determination of Cell Viability

MCF-10AT1 cells (2 × 10^4^/well) were plated in serum-free media (100 μL/well) in 84 wells of a 96-well plate. The plate was left overnight in a humidified incubator at 37 °C with 5% CO_2_ for adherence. The media were removed, and the wells were subjected to triplicate treatment with 100 μL of the previously described treatment media (as mentioned above) at *n* = 8 replicates. After 24–72 h of incubation, the CELLPRO-RO Roche Cell Viability and Proliferation Reagent, water-soluble tetrazolium salt (WST-1), was employed to assess cell viability according to the manufacturer’s protocol. 

### 4.5. Bromodeoxyuridine (BrdU) Cell Proliferation (Chemiluminescent) Assay

Cell proliferation was assessed using the Cell Signaling Technology BrdU Cell Proliferation Assay Kit (Danvers, MA, USA), adhering to the manufacturer’s protocol and established methodologies from previous studies [12]. The MCF-10AT1 cells were seeded at a 5 × 10^4^/well density into 84 wells (100 μL/well) of a 96-well plate and subjected to treatments outlined above for 12 and 24 h in triplicate experiments at *n* = 8 replicates. Post-treatment, the cells were placed in a humidified incubator at 37 °C, 5% CO_2_ for 24 h, then underwent fixation, primary and secondary antibody labeling, and luminal enhancer solution. Luminescence measurements at 450 nm were determined using the Bio Tek Synergy H1 Microplate Reader (Bio-Tek Instruments, Inc., Winooski, VT, USA).

### 4.6. Clonogenic Formation Assay

Cells were cultured in 5% dextran-coated charcoal-treated HS-DMEM/F12 media with the above-mentioned supplements. They were then seeded (2.5 × 10^2^/well) and allowed to incubate for 7 days at 37 °C with 5% CO_2_ in a six-well plate, facilitating adherence and proliferation. In the subsequent week, the cells underwent treatment with the previously described supplemented serum-free media in triplicate experiments with *n* = 3 replicates for 7 more days. Media changes with the respective treatments were administered for 5 days. The media was aspirated after 2 weeks, and the cells were fixed using a glutaraldehyde solution for 30 min and allowed to dry overnight. The next day, cells were stained with crystal violet for 30 min, rewashed, and left to dry overnight. The colonies were counted on the following day. 

### 4.7. Reactive Oxygen Species (ROS) Detection Assay

MCF-10AT1 cells (1 × 10^4^/well) were seeded in serum-free media into 84 wells (100 μL/well) of a 96-well plate and allowed to adhere overnight in a humidified incubator at 37 °C, 5% CO_2_. The reactive oxygen species determination protocol, set by the manufacturer’s instructions from BioVision Incorporated, was utilized for the ROS Detection Assay Kit. Briefly, pre-warmed ROS assay buffer was used to dilute the ROS (1000×) label to a final stock solution (1:1000). The adherent cells were washed in ROS assay buffer (100 μL), aspirated, and incubated for 45 min in 100 μL of diluted 1X ROS label solution. Then, the ROS label solution was aspirated. As described earlier, treatments (100 μL) were applied to each well in triplicate experiments at *n* = 8 for 12 and 24 h. Using 0.1% H_2_O_2_ as a positive control, fluorescence measurements at Ex/Em = 495/529 were conducted with the BioTek Synergy H1 Microplate Reader (Bio-Tek Instruments, Inc., Winooski, VT, USA). 

### 4.8. 8-Hydroxy-2-Deoxyguanosine (8-OHdG) Detection

Operating under the method applied in prior studies [12], 8-OHdG was identified and measured upon completion of the EpiQuik 8-OHdG DNA Damage Quantification Direct Kit (Colorimetric). 

### 4.9. Western Blot 

The cell pellets were obtained from untreated cells in media alone, 0.1% DMSO vehicle control, B[a]P (1 μM), DATS (40 μM), and CoTx (1 μM B[a]P combined with 40 μM DATS), respectively, following a 24 h treatment. A mixture of 0.5% TritonX-100 and a protease inhibitor cocktail was added to each pellet, and the Pierce BCA Protein Assay kit was used to determine the protein concentration. Each sample possessed 50 μg of protein, and the primary and secondary antibodies were used at a dilution of 1:1000. Following the incubation with the secondary antibody, the protein was identified, and a digital immunoblot was captured. The primary antibodies assessed included CYP1A1 (ab235185) obtained from Abcam, the Hypoxia Pathway Antibody Sampler Kit (#15792), AhR mAb (#83200), and loading control GAPDH mAb (#D16H11) or Vinculin mAb (#13901) purchased from Cell Signaling. 

### 4.10. Capillary Electrophoresis (Wes) Western Analysis 

The cell pellets were procured from untreated cells in media alone, 0.1% DMSO vehicle control, B[a]P (1 μM), DATS (40 μM), and CoTx (1 μM B[a]P combined with 40 μM DATS) following a 24 h treatment. A solution of 0.5% TritonX-100 mixed with a protease inhibitor cocktail was added to each pellet. Protein concentration was assessed using the Pierce BCA Protein Assay kit, with each sample comprising 2 mg/mL of protein for Wes analysis. Primary and secondary antibodies were used at a dilution of 1:125. Samples were prepared, heated, and loaded into the microplate, and then the Protein Standard Ladder, primary and secondary antibody, antibody diluent (blocking buffer), Streptavidin-HRP, wash buffer, and chemiluminescent solution were pipetted into the corresponding microplate wells. The microplate and capillary were then loaded into the device as directed by the manufacturer’s instructions (ProteinSimple, San Jose, CA, USA). The protein was identified upon completion of the capillary reaction and a digital immunoblot was captured. Thereafter, ProteinSimple SW Compass 6.2.0 software was used for the quantification and analysis of the digital image of the blots. Normalization of ProteinSimple WES™ data was accomplished through GAPDH. The primary antibodies examined were anti-Ogg1 (ab62826) and anti-DNA polymerase β (ab26343) purchased from Abcam, along with the loading control GAPDH mAb (#D16H11) from Cell Signaling. 

### 4.11. Statistical Analysis

All experiments were performed in triplicate (*n* = 3) with a minimum of three biological replicates. Analysis of all experimental data was performed using GraphPad Prism 9.0 software (San Diego, CA, USA). The results, presented as average values ± SEM, were assessed to identify significant differences employing one-way analysis of variance (ANOVA) and then Dunnett’s Multiple Comparison Test between the DMSO vehicle (*), B[a]P (#), and distinct treatment groups.

## 5. Conclusions

Our results indicate that DATS and CoTx may prevent B[a]P-induced carcinogenesis by attenuating cell proliferation, clonogenic formation, oxidative stress, DNA damage (generation of GC: TA transversion mutations), and the expression of proteins’ regulating metabolism and oxidative stress. Therefore, our findings suggest that garlic and its OSCs may have prophylactic effects and be an effective chemopreventive agent due to its anti-proliferative, antioxidant, antitumor, and anticancer abilities. Our findings uncover novel experimental evidence concerning the role of garlic organosulfide, DATS, in early transformed premalignant cells. Future studies must be performed to gain more insight into the role of garlic as a chemopreventive agent against the development of aggressive breast cancer phenotypes and fully decipher the precise mechanism by which DATS and OSCs elicit their effects.

## Data Availability

Data is contained within the article.
